# Supplementary material for: EPOP and MTF2 activate PRC2 activity through DNA-sequence specificity
Source: Proc Natl Acad Sci U S A. 2026 Feb 6;123(6):e2527303123. doi: 10.1073/pnas.2527303123 (PMC12890814; doi:10.1073/pnas.2527303123)
Supplement: Supplementary file 1 — Appendix 01 (PDF) [file pnas.2527303123.sapp.pdf]

Supporting Information for
EPOP and MTF2 Activate PRC2 Activity through DNA-sequence Specificity

Jeffrey Granat[1,2,3,9], Sanxiong Liu[1,2,4,5,6,9,*], Luis Popoca[1,2,7], Ozgur Oksuz[1,2,8], and Danny Reinberg[1,2,4,5,6,10,*]
[1]Howard Hughes Medical Institute, NYU Langone Health, New York, NY 10016, USA
[2]Department of Biochemistry and Molecular Pharmacology, NYU Langone Health, New York, NY 10016, USA
3Present address:
Columbia University Vagelos College of Physicians and Surgeons, New York, NY, 10032
Division of Hematology/Oncology, Department of Medicine, Columbia University Irving Medical Center, New York, NY, 10032
[4]Howard Hughes Medical Institute, Miller School of Medicine, University of Miami, Miami, FL 33136, USA
[5]Department of Human Genetics, Miller School of Medicine, University of Miami, Miami, FL 33136, USA
[6]Sylvester Comprehensive Cancer Center, Miller School of Medicine, University of Miami, Miami, FL 33136, USA
[7]Present address: Matter Bio, New York, NY 10014, USA
[8]Present address: NextRNA Therapeutics, Boston, MA 02135, USA
[9]These authors contributed equally
[10]Lead contact
*Sanxiong Liu, Danny Reinberg
Email: sxl4818@miami.edu (S.L.), dxr1274@miami.edu (D.R.)

This PDF file includes:

Figures S1 to S5

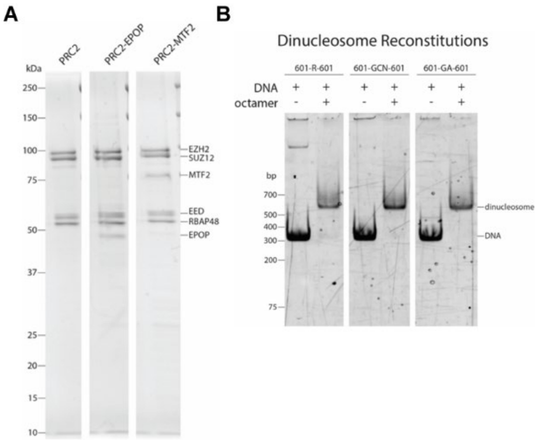

Figure S1. Quality control of purified PRC2 complexes and reconstituted dinucleosome substrates.

(A) Coomassie-stained SDS–PAGE of purified PRC2-core, PRC2–EPOP, and PRC2–MTF2 complexes used for HMT and EMSA assays. Bands corresponding to core subunits (EZH2, SUZ12, EED, RBAP48) and accessory factors (EPOP or MTF2) are indicated. (B) Agarose gel analysis of the 2×601-R, 2×601-GA, and 2×601-GCN DNA templates before and after dinucleosome reconstitution. DNA was visualized by ethidium bromide staining. Successful dinucleosome reconstitution is indicated by a mobility shift relative to free DNA for all three linker-sequence variants.

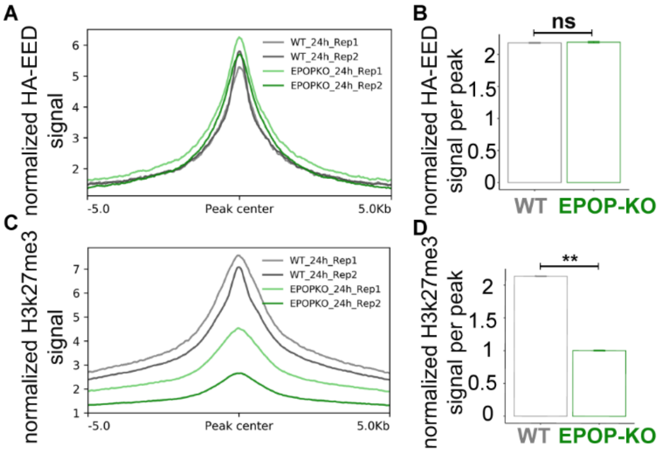

Figure S2. Quantification of HA-EED and H3K27me3 ChIP-seq signals, corresponding to Fig. 2.

(A and C) Profile plots for HA-EED (A) and H3K27me3 (C) ChIP-seq signal within ±5 kb of WT-defined peaks in WT and EPOPKO mESCs. (B and D) Quantification of HA-EED (B) and H3K27me3 (D) ChIP-seq signal across WT-defined peaks in WT and EPOPKO cells. Bars represent normalized ChIP-seq signal from individual replicates, with statistical comparison between WT and EPOPKO conditions.

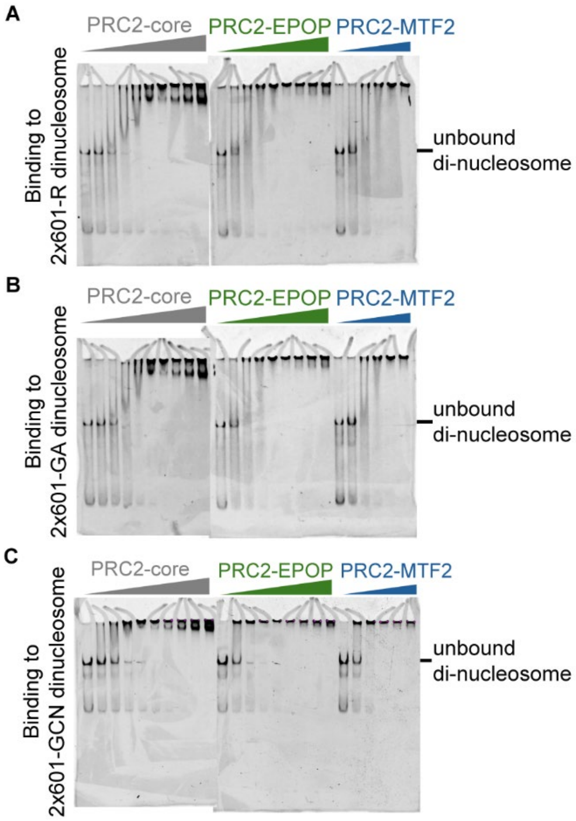

Figure S3. EMSAs resolved on acrylamide gels for PRC2–core, PRC2–EPOP, and PRC2–MTF2 binding to dinucleosomes with defined linker sequences, corresponding to Fig. 4. (A–C) EMSAs of PRC2–core, PRC2–EPOP, and PRC2–MTF2 incubated with dinucleosomes containing two Widom 601 positioning sequences separated by a 40 bp linker comprising either a random sequence [R; (A)], GA tandem repeats (B), or GCN tandem repeats (C). For each condition, half of the reactions shown in Figures 4A, C, and E were resolved on agarose gels (Figure 4), while the other half were resolved in parallel on acrylamide gels to provide increased detection sensitivity. DNA was visualized by SYBR Gold staining. These acrylamide gels were used for quantitative analysis and curve fitting shown in Figures 4B, D, and F.

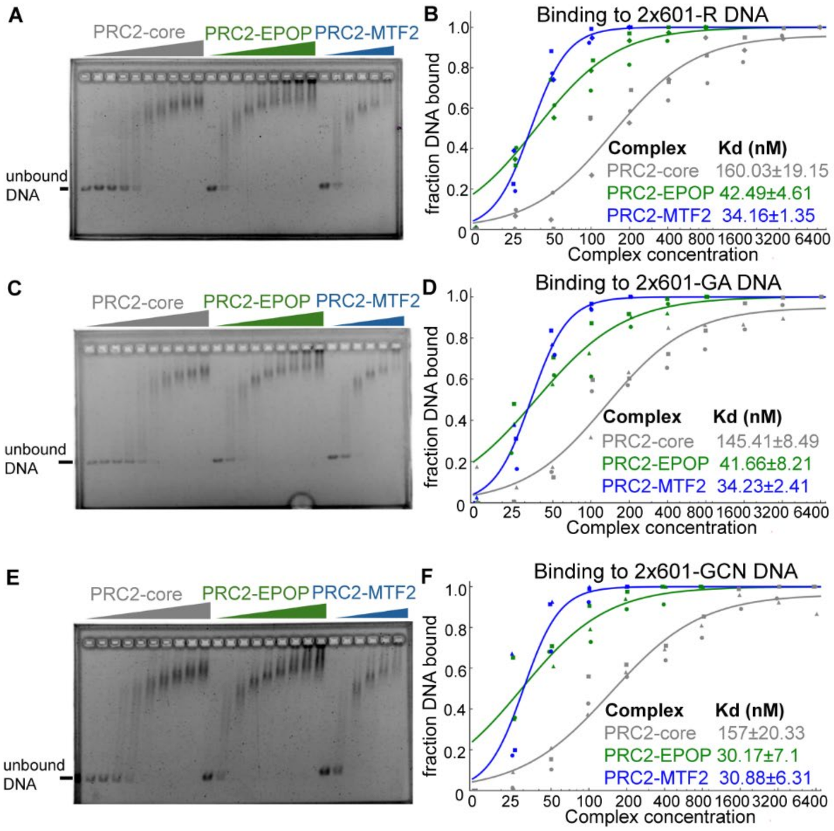

Figure S4. EPOP and MTF2 Enhance PRC2 Binding to Free DNA Independent of Linker Sequence, with MTF2 Conferring the Strongest Affinity.

(A, C, E) EMSAs examining binding between PRC2-core, PRC2–EPOP, or PRC2–MTF2 and free DNA with two Widom 601 positioning sequences separated by a 40 bp linker. Linker identities: random sequence [R; (A)], GA tandem repeats (C), or GCN tandem repeats (E). Reactions were incubated for 30 min and resolved on agarose gels; DNA was visualized by SYBR Gold staining. (B, D, F) Quantitative binding analysis for R- (B), GA- (D), and GCN- (F) linker DNA, measured from the other half of each reaction in A, C, and E, respectively, resolved on acrylamide gels for increased sensitivity. The corresponding acrylamide gels are shown in Fig. S5A–C. Data were fit with a sigmoidal binding function to calculate dissociation constants (Kd). *n* = 3 independent experiments.

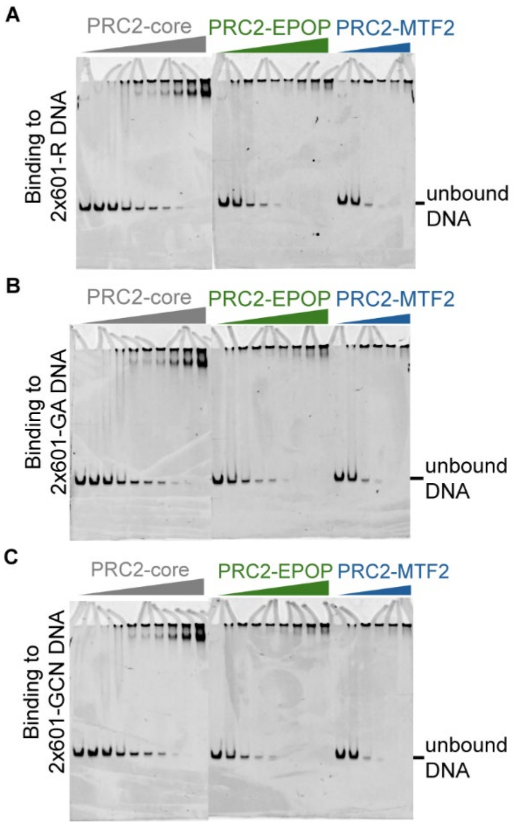

Figure S5. EMSAs resolved on acrylamide gels for PRC2–core, PRC2–EPOP, and PRC2–MTF2 binding to DNA with defined linker sequences, corresponding to Fig. S4.

(A–C) EMSAs of PRC2–core, PRC2–EPOP, and PRC2–MTF2 incubated with free DNA containing two Widom 601 positioning sequences separated by a 40 bp linker comprising either a random sequence [R; (A)], GA tandem repeats (B), or GCN tandem repeats (C). For each condition, half of the reactions shown in Figures S4A, C, and E were resolved on agarose gels (Fig. S4), while the other half were resolved in parallel on acrylamide gels to provide increased detection sensitivity. DNA was visualized by SYBR Gold staining. These acrylamide gels were used for quantitative analysis and curve fitting shown in Fig. S4B, D, and F.
